# Supplementary material for: A Likelihood Approach for Real-Time Calibration of Stochastic Compartmental Epidemic Models
Source: PLoS Comput Biol. 2017 Jan 17;13(1):e1005257. doi: 10.1371/journal.pcbi.1005257 (PMC5240920; doi:10.1371/journal.pcbi.1005257)
Supplement: S1 File — (TAR.GZ) [file pcbi.1005257.s014.tar.gz › HSPH_Online-SI-Revision/MSS10/n100-extreme/n100-extreme_NEW_table-8.pdf]

| | | | | | | | | | | | | | | | | | | | | | | | | | | | |
|---|---|---|---|---|---|---|---|---|---|---|---|---|---|---|---|---|---|---|---|---|---|---|---|---|---|---|---|
| 1 | R0 | {2.76052, 16.3913, 0.1} | {2.25278, 9.64751, 0.14} | {{1.47608, 4.02511, 0.}, {2.53075, 11.6597, 1.}} | {18.6093, 41.1425, 214.} | inf | 695. | 449.093 | {199.097, 583.067} | 35.6358 | 0.226746 | newly | 417. | 518.36 | {430., 617.} | 26.9736 | 0.102599 | Reff | 2.73137 | 2.23397 | {1.46825, 2.50917} | 18.4249 | ar | 92 090. | 82 481.5 | {57 102., 89 843.} | 10.4992 |
| 2 | R0 | {2.18202, 15.1734, 3.} | {1.24724, 2.86807, 0.} | {{1.22544, 2.5364, 0.}, {1.25474, 3.26445, 0.}} | {42.8399, 81.098, 100.} | inf | 2011. | 415.286 | {273.06, 565.383} | 79.3493 | 0.702768 | newly | 1160. | 1339.61 | {910., 1765.} | 26.1147 | 0.102883 | Reff | 2.10076 | 1.2207 | {1.20008, 1.22769} | 41.8926 | ar | 84 264. | 36 710.8 | {34 835., 38 389.} | 56.4336 |
| 3 | R0 | {2.57891, 12.5971, 1.} | {2.03137, 6.46906, 0.06} | {{1.737, 5.43578, 0.}, {2.26582, 8.12562, 0.}} | {21.7204, 48.9504, 98.} | inf | 340. | 217.654 | {120.7, 304.726} | 36.6062 | 0.22198 | newly | 303. | 433.89 | {296., 586.} | 47.4686 | 0.160999 | Reff | 2.5648 | 2.02263 | {1.72998, 2.25542} | 21.6267 | ar | 90 302. | 79 148.9 | {70 748., 85 843.} | 12.4841 |
| 4 | R0 | {2.5856, 9.28508, 0.1} | {2.16088, 7.56595, 0.3} | {{1.3618, 3.37285, 0.}, {2.80569, 10.6973, 1.}} | {21.2519, 32.3633, 354.} | inf | 8. | 15.9961 | {4.53727, 29.3869} | 117.54 | 0.31221 | newly | 20. | 30.56 | {7., 60.} | 90. | 0.300189 | Reff | 2.58508 | 2.16025 | {1.36143, 2.80464} | 21.2508 | ar | 90 312. | 74 286. | {37 240., 92 652.} | 19.1047 |
| 5 | R0 | {2.4406, 12.1341, 1.} | {1.29697, 3.90131, 0.22} | {{1.06738, 2.19254, 0.}, {1.44485, 4.02511, 1.}} | {46.9945, 70.7253, 100.} | inf | 184. | 44.1198 | {12.371, 71.9803} | 76.373 | 0.759006 | newly | 170. | 102.34 | {34., 185.} | 45.6118 | 0.338025 | Reff | 2.43279 | 1.29455 | {1.06564, 1.44229} | 46.9237 | ar | 88 597. | 36 552.4 | {13 601., 53 640.} | 58.7819 |
| 6 | R0 | {2.72155, 15.4094, 2.} | {2.72872, 16.5947, 1.42} | {{2.14747, 10.7247, 0.}, {2.93371, 18.9813, 2.}} | {8.7995, 18.7487, 29.} | inf | 411. | 482.972 | {307.994, 576.154} | 27.1498 | 0.110265 | newly | 302. | 296.75 | {250., 345.} | 10.4404 | 0.0463562 | Reff | 2.7028 | 2.70768 | {2.13565, 2.91066} | 8.69763 | ar | 91 682. | 91 008.1 | {83 441., 93 612.} | 3.2071 |
| 7 | R0 | {2.63781, 14.3935, 0.1} | {2.32194, 13.6368, 0.05} | {{1.93539, 9.63533, 0.}, {2.81891, 18.2812, 0.}} | {16.4691, 22.8232, 140.} | inf | 448. | 387.658 | {273.599, 535.223} | 23.7102 | 0.117774 | newly | 307. | 284.19 | {239., 327.} | 11.8078 | 0.0548692 | Reff | 2.61787 | 2.30544 | {1.92368, 2.79658} | 16.3812 | ar | 91 192. | 85 039.1 | {77 674., 92 685.} | 7.76138 |
| 8 | R0 | {2.82391, 16.104, 1.} | {2.29618, 11.166, 0.28} | {{1.93419, 8.56054, 0.}, {2.5684, 13.7324, 1.}} | {19.0545, 31.4252, 78.} | inf | 560. | 392.852 | {276.116, 495.412} | 29.9763 | 0.168139 | newly | 385. | 373.14 | {307., 443.} | 11.4494 | 0.0522957 | Reff | 2.79954 | 2.27999 | {1.92249, 2.5478} | 18.9288 | ar | 92 664. | 85 165.2 | {77 716., 90 159.} | 8.19252 |
| 9 | R0 | {2.80645, 14.6457, 0.1} | {2.33934, 13.6516, 0.7} | {{1.93539, 9.63533, 0.}, {2.93488, 18.8445, 1.}} | {19.1089, 26.9957, 668.} | inf | 226. | 208.381 | {124.126, 314.411} | 32.2863 | 0.154904 | newly | 148. | 150.18 | {120., 178.} | 12.6351 | 0.0550691 | Reff | 2.79573 | 2.3305 | {1.92926, 2.92167} | 19.0552 | ar | 92 567. | 84 738.6 | {77 695., 93 544.} | 9.03143 |
| 10 | R0 | {1.86213, 5.4271, 0.1} | {2.47461, 8.24168, 0.11} | {{1.74092, 4.66877, 0.}, {2.99092, 10.3908, 0.}} | {36.4402, 56.4275, 192.} | inf | 32. | 65.2402 | {21.516, 103.426} | 116.757 | 0.31776 | newly | 74. | 109.67 | {48., 176.} | 64.0676 | 0.216217 | Reff | 1.8607 | 2.47177 | {1.73975, 2.98658} | 36.3906 | ar | 75 362. | 85 561.4 | {70 872., 93 997.} | 18.0159 |
| 11 | R0 | {2.27349, 14.6338, 1.} | {1.26087, 3.49771, 0.04} | {{1.22544, 3.07157, 0.}, {1.28596, 3.98513, 0.}} | {44.5404, 76.0984, 98.} | inf | 1278. | 281.721 | {199.089, 368.78} | 77.9561 | 0.671107 | newly | 843. | 722.55 | {504., 941.} | 21.5718 | 0.108995 | Reff | 2.22036 | 1.24395 | {1.20888, 1.26915} | 43.9752 | ar | 85 805. | 38 101.1 | {34 192., 41 409.} | 55.5957 |
| 12 | R0 | {2.77261, 14.1894, 0.1} | {2.77728, 18.5672, 0.01} | {{2.56757, 17.005, 0.}, {2.93978, 19.9817, 0.}} | {7.66939, 33.8083, 108.} | inf | 206. | 199.089 | {156.875, 235.426} | 12.5616 | 0.0602784 | newly | 178. | 105.91 | {85., 125.} | 40.6236 | 0.23103 | Reff | 2.76385 | 2.76862 | {2.55943, 2.93071} | 7.64896 | ar | 92 508. | 91 820.9 | {89 834., 93 758.} | 2.32886 |
| 13 | R0 | {2.65402, 19.3935, 2.} | {1.66499, 7.95476, 0.82} | {{1.28596, 3.98513, 0.}, {1.77826, 9.06807, 1.}} | {37.2654, 58.9823, 60.} | inf | 1354. | 532.031 | {258.019, 703.806} | 60.7067 | 0.438722 | newly | 658. | 618.65 | {526., 720.} | 11.6884 | 0.0560825 | Reff | 2.59651 | 1.64226 | {1.27126, 1.75425} | 36.7512 | ar | 91 184. | 65 231.5 | {39 941., 72 958.} | 28.4617 |
| 14 | R0 | {1.75297, 4.45354, 0.1} | {1.19469, 2.35039, 0.03} | {{1.06738, 2.19254, 0.}, {1.39162, 2.5364, 0.}} | {31.8478, 47.2242, 126.} | inf | 18. | 12.0302 | {3.38413, 20.9961} | 46.8837 | 0.314562 | newly | 29. | 56.8 | {10., 106.} | 131.586 | 0.350318 | Reff | 1.75187 | 1.194 | {1.06674, 1.39067} | 31.8441 | ar | 71 263. | 21 901.2 | {62., 49 957.} | 69.2671 |
| 15 | R0 | {1.99928, 7.1501, 0.1} | {2.4002, 12.8303, 1.25} | {{1.74092, 5.83416, 0.}, {2.98125, 18.9406, 2.}} | {25.2775, 85.8138, 1202.} | inf | 97. | 130.468 | {57.4399, 196.029} | 51.7623 | 0.193949 | newly | 168. | 107.75 | {74., 142.} | 35.9821 | 0.206909 | Reff | 1.99548 | 2.39465 | {1.73848, 2.97307} | 25.2163 | ar | 79 602. | 85 383.3 | {71 459., 93 889.} | 12.0092 |
| 16 | R0 | {2.67398, 10.3134, 1.} | {2.34849, 14.6528, 0.29} | {{1.81647, 9.63533, 0.}, {2.88523, 18.8857, 1.}} | {16.4735, 45.5459, 85.} | inf | 44. | 29.729 | {13.2086, 47.2037} | 37.5855 | 0.239097 | newly | 54. | 20.59 | {9., 33.} | 61.9074 | 0.471552 | Reff | 2.67219 | 2.34722 | {1.81568, 2.88356} | 16.466 | ar | 91 289. | 84 742.1 | {73 630., 93 264.} | 8.29516 |
| 17 | R0 | {2.44634, 16.3226, 0.1} | {2.85796, 17.4615, 0.} | {{2.46794, 14.1192, 0.}, {2.96797, 18.4042, 0.}} | {16.8261, 12.917, 100.} | inf | 1288. | 1620.03 | {1283.23, 1793.63} | 26.5369 | 0.0997592 | newly | 777. | 942.91 | {875., 1003.} | 21.3526 | 0.0834677 | Reff | 2.39443 | 2.78746 | {2.41541, 2.8945} | 16.4145 | ar | 88 710. | 92 774.4 | {88 872., 93 963.} | 4.58898 |
| 18 | R0 | {2.02168, 10.1676, 0.1} | {2.89658, 18.3595, 0.03} | {{2.88523, 18.2127, 0.}, {2.96978, 18.8857, 0.}} | {43.276, 80.5689, 124.} | inf | 354. | 652.696 | {598.765, 711.721} | 84.5994 | 0.264409 | newly | 351. | 359.98 | {317., 404.} | 7.68091 | 0.0329589 | Reff | 2.00733 | 2.8673 | {2.85493, 2.93951} | 42.8417 | ar | 80 299. | 93 158.3 | {93 071., 93 930.} | 16.0142 |
| 19 | R0 | {2.89969, 15.009, 0.1} | {2.29214, 10.9342, 0.08} | {{1.737, 5.43578, 0.}, {2.7499, 15.6124, 0.}} | {21.0464, 30.3491, 166.} | inf | 224. | 155.916 | {64.1833, 233.61} | 32.9704 | 0.21974 | newly | 179. | 158.86 | {112., 199.} | 22.838 | 0.116354 | Reff | 2.88972 | 2.28557 | {1.73405, 2.74013} | 21.0022 | ar | 93 349. | 83 337. | {70 865., 92 104.} | 10.7457 |
| 20 | R0 | {2.68933, 7.34928, 0.1} | {2.11886, 10.4667, 0.64} | {{1.25474, 4.02511, 0.}, {2.86501, 17.1903, 2.}} | {23.6392, 58.7621, 652.} | inf | 3. | 9.17301 | {2.19768, 17.4773} | 217.277 | 0.419303 | newly | 7. | 11.38 | {2., 26.} | 113.714 | 0.391254 | Reff | 2.68909 | 2.11852 | {1.25459, 2.8645} | 23.6407 | ar | 91 574. | 69 869.7 | {26., 92 881.} | 24.2674 |
| 21 | R0 | {2.15352, 9.28074, 0.1} | {2.38136, 11.7851, 1.17} | {{1.53501, 4.02511, 0.}, {2.8833, 17.7348, 2.}} | {21.6857, 47.5313, 1134.} | inf | 131. | 152.282 | {63.4667, 218.976} | 41.5064 | 0.204055 | newly | 151. | 142.95 | {91., 196.} | 24.1788 | 0.126262 | Reff | 2.14827 | 2.37479 | {1.53201, 2.87432} | 21.6293 | ar | 83 845. | 84 038.6 | {60 345., 93 326.} | 11.1751 |
| 22 | R0 | {2.21953, 9.04387, 0.1} | {1.81874, 7.32559, 0.78} | {{1.24751, 2.5364, 0.}, {2.688, 15.4278, 3.}} | {27.0474, 58.5382, 812.} | inf | 114. | 54.5246 | {14.4313, 103.432} | 53.6527 | 0.433656 | newly | 136. | 94.42 | {36., 150.} | 46.8235 | 0.29246 | Reff | 2.21565 | 1.81649 | {1.24655, 2.683} | 27.0173 | ar | 84 856. | 65 432.5 | {36 467., 91 318.} | 26.3001 |
| 23 | R0 | {2.21987, 14.5347, 1.} | {1.35144, 4.28465, 0.88} | {{1.25474, 3.26445, 0.}, {1.454, 4.94504, 2.}} | {39.1207, 70.5213, 104.} | inf | 1324. | 377.058 | {245.628, 509.34} | 71.5213 | 0.56284 | newly | 822. | 818.04 | {621., 1012.} | 16.0049 | 0.0716353 | Reff | 2.16759 | 1.33234 | {1.23714, 1.43161} | 38.5338 | ar | 85 067. | 46 298.2 | {37 486., 55 766.} | 45.5744 |
| 24 | R0 | {2.84268, 17.9659, 1.} | {1.43346, 4.29788, 0.07} | {{1.24751, 2.5364, 0.}, {1.47608, 4.06661, 0.}} | {49.5735, 76.0776, 95.} | inf | 799. | 161.151 | {38.4266, 235.299} | 79.8309 | 0.844658 | newly | 439. | 364.04 | {91., 604.} | 36.0957 | 0.236564 | Reff | 2.80769 | 1.42459 | {1.24054, 1.46906} | 49.2612 | ar | 92 836. | 49 109.1 | {35 354., 57 628.} | 47.1012 |
| 25 | R0 | {2.52706, 10.3679, 0.1} | {1.16262, 2.36358, 0.09} | {{1.06738, 2.19254, 0.}, {1.24751, 2.5364, 0.}} | {53.9932, 77.2029, 180.} | inf | 97. | 25.1413 | {5.26598, 46.1266} | 74.0811 | 0.691141 | newly | 97. | 95.98 | {13., 186.} | 54.5567 | 0.324087 | Reff | 2.52279 | 1.16148 | {1.06622, 1.24647} | 53.9603 | ar | 89 623. | 21 833.7 | {108., 37 744.} | 75.6383 |
| 26 | R0 | {2.62835, 19.1088, 1.} | {1.6641, 7.36057, 0.51} | {{1.22544, 3.07157, 0.}, {2.1245, 12.2645, 1.}} | {36.7779, 61.4807, 59.} | inf | 1699. | 644.563 | {202.518, 1108.26} | 62.0622 | 0.528177 | newly | 814. | 819.24 | {621., 964.} | 13.6978 | 0.0602134 | Reff | 2.55757 | 1.63488 | {1.21022, 2.08205} | 36.2054 | ar | 90 908. | 58 450.2 | {35 085., 82 971.} | 35.7269 |
| 27 | R0 | {2.76831, 19.4155, 2.} | {2.58014, 15.5153, 1.54} | {{1.44762, 4.00571, 0.}, {2.97157, 19.3547, 2.}} | {16.7831, 20.088, 23.} | inf | 2186. | 1904.92 | {481.62, 2451.08} | 25.4779 | 0.171763 | newly | 1084. | 1213.16 | {1139., 1286.} | 12.7897 | 0.0517065 | Reff | 2.67358 | 2.49466 | {1.42392, 2.86594} | 16.3714 | ar | 92 175. | 85 294.2 | {54 458., 93 985.} | 9.95835 |
| 28 | R0 | {2.96609, 16.8903, 1.} | {2.37145, 9.24317, 0.7} | {{1.47608, 3.83841, 0.}, {2.8186, 12.1249, 1.}} | {20.2275, 45.2753, 46.} | inf | 591. | 335.437 | {145.024, 482.108} | 43.2533 | 0.282973 | newly | 383. | 445.21 | {333., 559.} | 23.3185 | 0.0890226 | Reff | 2.94132 | 2.35717 | {1.47074, 2.80036} | 20.0624 | ar | 94 024. | 83 434.6 | {57 117., 92 715.} | 11.2727 |
| 29 | R0 | {2.17046, 14.39, 0.1} | {1.25442, 3.15505, 0.04} | {{1.22544, 2.5364, 0.}, {1.28596, 3.98513, 0.}} | {42.2051, 78.0747, 136.} | inf | 1169. | 279.634 | {176.458, 365.683} | 76.0792 | 0.655741 | newly | 743. | 828.94 | {517., 1197.} | 27.467 | 0.125537 | Reff | 2.12193 | 1.23752 | {1.20911, 1.268} | 41.6797 | ar | 84 001. | 37 051.9 | {33 951., 40 828.} | 55.8912 |
| 30 | R0 | {2.88772, 12.4647, 1.} | {1.42262, 3.74591, 0.26} | {{1.06738, 2.19254, 0.}, {1.88643, 4.66877, 1.}} | {50.7354, 71.2224, 98.} | inf | 43. | 19.3918 | {4.26329, 35.3352} | 60.9517 | 0.494759 | newly | 59. | 64.1 | {12., 107.} | 69.6949 | 0.318267 | Reff | 2.88584 | 1.42201 | {1.067, 1.88583} | 50.7248 | ar | 93 273. | 40 597.3 | {87., 76 102.} | 56.4748 |
| 31 | R0 | {2.46831, 8.14308, 0.1} | {2.02407, 9.39461, 0.65} | {{1.28596, 4.02511, 0.}, {2.8702, 15.4074, 2.}} | {24.293, 42.8215, 656.} | inf | 13. | 5.61509 | {2.24721, 9.27433} | 59.8241 | 0.452004 | newly | 16. | 17.07 | {1., 18.} | 118.188 | 0.555953 | Reff | 2.46792 | 2.02389 | {1.28589, 2.86994} | 24.2904 | ar | 88 762. | 66 324.3 | {12., 93 166.} | 27.175 |
| 32 | R0 | {1.93236, 7.38987, 0.1} | {2.81325, 9.78757, 0.18} | {{2.68628, 8.24535, 0.}, {2.99092, 10.6536, 1.}} | {48.9444, 41.3624, 256.} | inf | 96. | 215.392 | {160.318, 277.522} | 127.6 | 0.350781 | newly | 158. | 334.11 | {227., 405.} | 111.462 | 0.298457 | Reff | 1.92828 | 2.80384 | {2.67713, 2.98018} | 48.7632 | ar | 77 654. | 90 759.4 | {91 388., 93 976.} | 21.0403 |
| 33 | R0 | {2.82251, 17.6954, 1.} | {1.89574, 7.84188, 0.68} | {{1.44762, 3.83841, 0.}, {2.688, 14.8279, 2.}} | {32.8349, 55.6841, 88.} | inf | 586. | 265.596 | {93.6034, 500.791} | 54.6764 | 0.426578 | newly | 325. | 357.09 | {257., 464.} | 20.5631 | 0.0839791 | Reff | 2.7977 | 1.8844 | {1.43995, 2.66598} | 32.6446 | ar | 92 745. | 69 977.2 | {53 250., 91 474.} | 24.5488 |
| 34 | R0 | {1.87083, 12.3994, 1.} | {1.49581, 6.41361, 0.52} | {{1.22544, 3.07157, 0.}, {2.35866, 16.9062, 2.}} | {29.4331, 61.3598, 92.} | inf | 1336. | 716.098 | {291.715, 1966.77} | 63.2134 | 0.440695 | newly | 975. | 984.86 | {748., 1187.} | 14.0103 | 0.0624603 | Reff | 1.81905 | 1.46123 | {1.20431, 2.27829} | 28.7667 | ar | 76 282. | 50 249.7 | {34 792., 87 161.} | 39.28 |
| 35 | R0 | {2.95779, 16.3003, 1.} | {2.34876, 10.755, 0.14} | {{1.74592, 5.83416, 0.}, {2.53075, 12.5037, 1.}} | {20.5908, 34.2379, 90.} | inf | 233. | 182.636 | {123.258, 237.835} | 23.5551 | 0.130795 | newly | 159. | 192.49 | {140., 242.} | 26.5975 | 0.0985212 | Reff | 2.94682 | 2.34112 | {1.74194, 2.52158} | 20.5543 | ar | 93 873. | 85 859.4 | {71 710., 89 838.} | 8.5366 |
| 36 | R0 | {2.18718, 14.261, 3.} | {1.55439, 6.60625, 1.01} | {{1.22544, 2.5364, 0.}, {2.64373, 19.7855, 4.}} | {36.7056, 67.8662, 80.3333} | inf | 1258. | 545.976 | {173.552, 1568.5} | 66.2824 | 0.552594 | newly | 857. | 788.72 | {544., 984.} | 18.4177 | 0.0873636 | Reff | 2.13862 | 1.52813 | {1.21026, 2.57634} | 36.1696 | ar | 84 379. | 50 007.3 | {34 480., 90 905.} | 43.6454 |
| 37 | R0 | {2.44912, 9.31118, 0.1} | {1.91748, 7.37434, 0.76} | {{1.06738, 2.19254, 0.}, {2.95376, 15.278, 2.}} | {32.5047, 57.664, 780.} | inf | 41. | 35.8894 | {4.08839, 73.3621} | 52.5563 | 0.345304 | newly | 54. | 65.78 | {12., 114.} | 61.7037 | 0.257944 | Reff | 2.44728 | 1.916 | {1.0668, 2.95026} | 32.4922 | ar | 88 442. | 56 479.4 | {161., 93 580.} | 39.5317 |
| 38 | R0 | {2.05949, 5.72514, 0.1} | {1.30699, 2.44678, 0.05} | {{1.06738, 1.89883, 0.}, {1.65456, 2.5364, 0.}} | {36.9854, 62.108, 146.} | inf | 11. | 17.9275 | {3.24599, 31.1182} | 98.9487 | 0.325948 | newly | 26. | 115.04 | {12., 284.} | 364.231 | 0.5316 | Reff | 2.05842 | 1.3062 | {1.06677, 1.65363} | 36.9895 | ar | 81 587. | 33 993.6 | {89., 67 101.} | 58.5313 |
| 39 | R0 | {1.75505, 8.52404, 0.1} | {1.67666, 6.99698, 0.44} | {{1.24751, 2.5364, 0.}, {2.93488, 18.8445, 1.}} | {28.0144, 61.0488, 460.} | inf | 489. | 445.501 | {86.6202, 1159.95} | 61.8179 | 0.354993 | newly | 574. | 654.1 | {289., 1025.} | 31.878 | 0.136225 | Reff | 1.73539 | 1.6566 | {1.23636, 2.87872} | 27.6924 | ar | 70 925. | 56 737.5 | {35 940., 93 338.} | 34.0001 |
| 40 | R0 | {2.24724, 13.7861, 1.} | {1.25208, 2.76715, 0.} | {{1.22544, 2.5364, 0.}, {1.25474, 3.26445, 0.}} | {44.2839, 79.928, 100.} | inf | 1281. | 248.832 | {153.349, 352.088} | 80.5752 | 0.753136 | newly | 906. | 872.22 | {483., 1288.} | 27.5364 | 0.149168 | Reff | 2.19627 | 1.2366 | {1.21053, 1.239} | 43.6954 | ar | 85 257. | 36 880.7 | {34 764., 38 287.} | 56.7417 |
| 41 | R0 | {1.77635, 8.62406, 0.1} | {1.2665, 3.03164, 0.} | {{1.22544, 2.5364, 0.}, {1.44762, 3.98513, 0.}} | {28.7019, 64.8467, 100.} | inf | 634. | 204.38 | {99.53, 303.294} | 67.7633 | 0.542097 | newly | 682. | 635.55 | {350., 914.} | 28.934 | 0.158667 | Reff | 1.75212 | 1.25465 | {1.21388, 1.43168} | 28.3926 | ar | 72 941. | 38 470.4 | {33 785., 53 485.} | 47.2582 |
| 42 | R0 | {1.80397, 5.4932, 1.} | {1.44399, 4.31039, 0.34} | {{1.06738, 2.19254, 0.}, {2.31035, 12.5436, 2.}} | {29.9963, 57.0818, 104.} | inf | 36. | 31.701 | {9.97128, 63.5859} | 49.7953 | 0.274489 | newly | 83. | 86.82 | {29., 157.} | 51.6386 | 0.236944 | Reff | 1.80242 | 1.44274 | {1.06659, 2.30757} | 29.9852 | ar | 73 232. | 43 314.7 | {11 505., 86 600.} | 46.1762 |
| 43 | R0 | {2.83096, 16.7077, 0.1} | {1.71223, 5.42424, 0.07} | {{1.47608, 4.02511, 0.}, {1.74592, 5.83416, 0.}} | {39.5178, 67.5345, 158.} | inf | 491. | 199.03 | {135.203, 263.693} | 59.4645 | 0.408463 | newly | 311. | 413.49 | {276., 539.} | 38.5691 | 0.139136 | Reff | 2.80905 | 1.70395 | {1.47017, 1.73839} | 39.3408 | ar | 92 740. | 69 129.3 | {56 853., 71 521.} | 25.4591 |
| 44 | R0 | {2.73678, 11.6021, 1.} | {2.70421, 8.89001, 0.11} | {{1.94705, 4.66877, 0.}, {2.99092, 10.5991, 0.}} | {10.3942, 23.3759, 93.} | inf | 128. | 111.67 | {59.1705, 156.198} | 23.7151 | 0.130402 | newly | 125. | 192.39 | {117., 291.} | 58.856 | 0.185308 | Reff | 2.73147 | 2.69933 | {1.9453, 2.98434} | 10.3872 | ar | 92 003. | 90 213.2 | {78 978., 93 947.} | 4.13063 |
| 45 | R0 | {2.14235, 6.98601, 0.1} | {2.38371, 13.1827, 0.36} | {{1.94705, 4.08009, 0.}, {2.76795, 18.4249, 1.}} | {16.8247, 101.869, 394.} | inf | 49. | 38.1305 | {13.6736, 57.9576} | 33.9867 | 0.206925 | newly | 74. | 41.02 | {15., 73.} | 65.9459 | 0.436738 | Reff | 2.14079 | 2.38219 | {1.94523, 2.76593} | 16.8238 | ar | 82 974. | 85 380.3 | {78 237., 92 258.} | 8.66991 |
| 46 | R0 | {2.23409, 9.21289, 0.1} | {2.45342, 12.0046, 0.12} | {{1.74592, 5.83416, 0.}, {2.96947, 15.963, 1.}} | {17.6026, 42.7941, 196.} | inf | 58. | 63.422 | {27.8177, 86.6455} | 32.665 | 0.158047 | newly | 78. | 60.17 | {36., 85.} | 30.6538 | 0.171268 | Reff | 2.23183 | 2.45075 | {1.74502, 2.96589} | 17.5852 | ar | 85 163. | 86 466.3 | {71 421., 93 863.} | 8.10743 |
| 47 | R0 | {2.12258, 8.15265, 0.1} | {2.53434, 7.45644, 0.26} | {{1.67287, 3.37285, 0.}, {2.99092, 10.3743, 1.}} | {25.9801, 25.8431, 320.} | inf | 73. | 95.0596 | {42.8858, 138.086} | 49.7956 | 0.187616 | newly | 100. | 232.65 | {120., 342.} | 133.63 | 0.305868 | Reff | 2.11978 | 2.53033 | {1.6715, 2.98522} | 25.944 | ar | 82 690. | 87 190.1 | {68 039., 93 888.} | 11.101 |
| 48 | R0 | {2.71771, 10.4636, 0.1} | {2.52324, 8.79848, 0.2} | {{1.81768, 5.43578, 0.}, {2.9751, 10.6536, 1.}} | {13.391, 17.8968, 268.} | inf | 20. | 36.4097 | {13.1303, 55.7754} | 97.3479 | 0.305402 | newly | 23. | 57.67 | {20., 96.} | 158.913 | 0.381689 | Reff | 2.71654 | 2.5217 | {1.81695, 2.97301} | 13.3837 | ar | 91 716. | 86 544.8 | {73 611., 93 783.} | 7.23596 |
| 49 | R0 | {2.44369, 6.66477, 0.1} | {1.936, 4.28414, 0.08} | {{1.22544, 2.37684, 0.}, {2.77593, 6.23525, 0.}} | {26.9521, 41.1329, 170.} | inf | 7. | 10.5694 | {3.06466, 19.02} | 78.3825 | 0.271625 | newly | 14. | 64.62 | {8., 140.} | 376.143 | 0.513963 | Reff | 2.44335 | 1.93564 | {1.22534, 2.7752} | 26.9517 | ar | 88 827. | 64 756.9 | {32., 92 200.} | 28.7626 |
| 50 | R0 | {2.77383, 17.9775, 0.1} | {2.94019, 18.6502, 0.} | {{2.88523, 18.2127, 0.}, {2.96978, 18.8857, 0.}} | {5.9974, 3.74166, 100.} | inf | 1401. | 1410.13 | {1335.73, 1484.87} | 3.47067 | 0.0149666 | newly | 799. | 765.48 | {719., 813.} | 5.09387 | 0.0229364 | Reff | 2.71353 | 2.87599 | {2.82252, 2.90606} | 5.98705 | ar | 92 334. | 93 620.2 | {93 139., 93 976.} | 1.39298 |
